# Comparison between effects of HydroCoil Embolic System and ordinary coil on large- and medium-sized intracranial aneurysms

**DOI:** 10.12669/pjms.296.3640

**Published:** 2013

**Authors:** De Wei, Chenbin Wei, Shaosong Huang, Qiuping Lin, Yiling Wang

**Affiliations:** 1 Dr. De Wei, Department of Neurosurgery, Provincial Clinical College of Fujian Medical University, Fuzhou 350001, P. R. China.; 2 Dr. Chenbin Wei, Provincial Clinical College of Fujian Medical University, Fuzhou 350001, P. R. China.; 3 Dr. Shaosong Huang, Department of Neurosurgery, Provincial Clinical College of Fujian Medical University, Fuzhou 350001, P. R. China.; 4 Dr. Qiuping Lin, Provincial Clinical College of Fujian Medical University, Fuzhou 350001, P. R. China.; 5 Dr. Yiling Wang, Provincial Clinical College of Fujian Medical University, Fuzhou 350001, P. R. China.

**Keywords:** Intracranial aneurysm, HydroCoil

## Abstract

***Objective:*** To explore the treatment effects of HydroCoil Embolic System (HES) on large- and medium-sized intracranial aneurysms.

***Methodology:*** Fifty cases of intracranial aneurysm patients were retrospectively analyzed, in which 27 and 23 cases were treated with HES (n=27) and ordinary coils (n=23), respectively. All the patients were followed up for two years.

***Results:*** The 27 cases (54%, 27/50) treated with HES include 23 cases of densely packed occlusion (46%, 23/50) and 4 cases of subtotal occlusion (8%, 4/50). The 23 cases (46%, 23/50) treated with ordinary coils include 15 cases of densely packed occlusion (30%, 15/50) and 8 cases of subtotal occlusion (16%, 8/50).

***Conclusion:*** HydroCoil Embolic System (HES) may increase the ratio of densely packed occlusion and prevent the recurrence of large- and medium-sized intracranial aneurysms.

## INTRODUCTION

Interventional embolization treatment of intracranial aneurysm has been substantially improved owing to the newly designed embolic materials in recent years. HydroCoil Embolic System (HES) developed by Micro-Vention consists of a traditional platinum coil coupled with an expandable and nonabsorbable hydrogel, which increases the volume ratio of the material when placed into a physiologic environment, thereby leading to the more densely packed occlusion of aneurysm body and aneurysm neck. However, wide-necked large- and medium-sized aneurysms are prone to remaining and recurring due to the limitations of interventional therapy. In this paper, we compared the treatment effects of stent-assisted or balloon-assisted HES and ordinary coil on large- and medium-sized aneurysms.

## METHODOLOGY

***General Information: ***Fifty cases were selected from the intracranial aneurysm inpatients in Department of Neurosurgery, Provincial Clinical College of Fujian Medical University (January 2006 - October 2010). *Diagnosis criteria:* All the patients had been diagnosed by spiral CT angiography (CTA), and some of them had received brain magnetic resonance imaging (MRI) and magnetic resonance angiography (MRA) before surgery. Wide-necked intracranial aneurysms include absolute and relative ones, referring to the neck width ≥ 4mm and neck to body ratio higher than 1/2.^1^ The diameters (D) of medium-, large- and giant-sized aneurysms are 0.5 cm≤ D≤ 1.5cm, 1.5 cm≤ D≤ 2.5cm, and D> 2.5cm, respectively.

The above cases were further diagnosed by digital subtraction angiography (DSA) as large- and medium-sized aneurysms. Patients with aneurysms diametered lower than 0.5 cm were excluded from this study. All the cases were divided into HES group (27 cases) and ordinary coil group (23 cases). The HES group (Male: 12 cases, Female: 15 cases; aged 42-76, average: 52.3) received HydroCoil embolization. Aneurysms in 25 out of 27 cases had ruptured, which manifested clinically as headache, vomiting and consciousness disturbances. The patients were urgently hospitalized owing to the subarachnoid hemorrhage or hematoma indicated by brain CT scan. The two cases with unruptured aneurysms that manifested clinically as undefined eyelid drooping and diplopia-associated headache were hospitalized after the diagnoses by outpatient or Ophthalmology dept. 12 and 15 cases were medium- and large-sized aneurysms.

The ordinary coil group (Male: 9 cases, Female: 14 cases; aged 38-72, average: 51.4) received ordinary coil embolization. Thirteen and 10 cases were medium- and large-sized aneurysms. The Hunt-Hess grades of the patients included 6 cases of Grade I, 12 cases of Grade II-III, 4 cases of Grade IV and one case of Grade V. The aneurysms were found in different body areas (internal carotid-posterior communicating aneurysm: 8 cases, carotid-ophthalmic aneurysm: four cases, carotid-anterior choroidal aneurysm: one case, anterior communicating aneurysm: five cases, middle cerebral aneurysm: Two cases, vertebral aneurysm: 2 cases, posteriorcerebral aneurysm: 1 case). The ages and the aneurysm characteristics of the two patient groups did not differ significantly.

***Methods: ***By means of digital subtraction especially 3D imaging, the intracranial aneurysms images were analyzed, the 3D characteristics of the aneurysms, the positions of aneurysm necks and bodies, and the relationships between aneurysms and tumor-carrying arteries or neighboring arteries were clarified, the aneurysm spatial configuration was described, and different personalized assistance methods were designed. The embolic extents are classified into densely packed occlusion (unvisualized tumor neck and tumor body), neck tumor residue (unvisualized tumor body, partially or completely visualized tumor neck) and partial occlusion (visualized tumor body). V_aneurysm_ = 4/3π×length/2×width/2×height/2. V_coil_ = sectional area × length. Packing density = V_coil_/V_aneurysm_.

## RESULTS

***Embolic results: ***Twenty-seven out of fifty cases (54%, 27/50) were treated with HES, including 23 cases of densely packed occlusion (46%, 23/50) and 4 cases of subtotal occlusion (8%, 4/50). The 23 cases (46%, 23/50) treated with ordinary coils included 15 cases of densely packed occlusion (30%, 15/50) and 8 cases of subtotal occlusion (16%, 8/50). In the 25 cases of medium-sized cerebral aneurysms, the 15 cases of densely packed occlusion consist of 10 cases of HydroCoil embolization (40%, 10/25) and 5 cases of ordinary coil embolization (20%, 5/25). The 10 cases of subtotal occlusion include 2 cases of HydroCoil embolization and 8 cases of ordinary coil embolization. In the 25 cases of large-sized cerebral aneurysms, the 13 cases of densely packed occlusion consist of 10 cases of HydroCoil embolization (42%, 8/19) and 5 cases of ordinary coil embolization (11%, 2/19). The 12 cases of subtotal occlusion include 5 cases of HydroCoil embolization and 7 cases of ordinary coil embolization. There were no partial occlusion or occlusion failure cases. The average inpatient duration of the two groups did not differ significantly. HES may increase the densely packed occlusion rate and decrease the recurrence rate of large- and medium-sized intracranial aneurysms. The treatment effects of the two groups differed significantly (P<0.05).

***Complications: ***The aneurysm of one out of the 27 patients receiving HydroCoil embolization ruptured during surgery (1/27, 3.7%), leading postoperative limb movement disorder after conservative treatment. Two out of the 23 patients receiving ordinary coil embolization suffered from vascular spasm (1/23, 4.3%). Although the complication was relieved after injecting papaverine by micro-catheter, the contralateral anterior cerebral arteries were visualized faintly. Besides, the anterior cerebral arteries were well-visualized. The patients had no clinical symptoms. The complications of the two groups did not statistically differ significantly. No patients died.

***Follow-up: ***Only one of the 27 cases receiving HydroCoil embolization suffered from basilar aneurysm recurrence during the postoperative 2-year follow-up. However, 3 and 2 of the 23 cases receiving ordinary coil embolization suffered from aneurysm recurrence and re-rupture, respectively. The recurrent rates of aneurysms in the HES group were significantly lower than those in the ordinary coil group (P<0.05).

## DISCUSSION

Subarachnoid hemorrhage tests have demonstrated that interventional endovascular embolization can apparently reduce mortality rate as an effective substitute for surgical clipping. Besides, embolization decreases the absolute risk by 7.4% and prevents epilepsy.^2^ However, the wide-necked large- or giant-sized aneurysms cannot be effectively and continuously embolized after endovascular embolization, leading to the re-rupture and hemorrhage of patients. The occlusion of intracranial aneurysms also requires thrombosis. Nevertheless, some unstable thrombosis may dissolve, which frequently induces the recurrence of aneurysm.^3^ Therefore, particular attention should be paid to ensuring treatment security while reducing recurrence rate. The densely packed ordinary platinum coils angiographically only account for 20-30% of the occlusion actually, which may easily result in aneurysm recurrence.^1^

HES consists of an outer HydroCoil and an inner expandable Hydrosoft. The biocompatible Hydrogel coating is an acrylic acid copolymer that expands in water. The copolymer begins to absorb water and expand after being placed in blood for five minutes owing to carboxyl deprotonation. The packing density then increases exponentially until the copolymer completely expands (20 min). The expanded Hydrogel automatically fills the gap between coils. As a result, aneurysm recanalization is prevented by dense packing instead of thrombosis. Hydrogel does not decompose in human body and is not subject to physiological thrombolysis and continuous arterial pulse.^3^^-^^5^

Compared to ordinary coils, the smooth muscle cells of the aneurysm embolized by HES develop more rapidly, so that the arterial wall across the aneurysm was continuously reconstructed much more easily. Hydrosoft is a novel interventional material, consisting of a platinum coil and an expandable hydrogel core. The flexible platinum coil hardly triggers tube dislodgment and is free from operating time restriction, unwinding and vapor preparation. On the other hand, the hydrogel that covers the aneurysm neck can reduce the recurrence rate and fill a volume 70% higher than the ordinary platinum coil does. Moreover, the non-toxic hydrogel is also biologically inert. Microvention-Microplex products can significantly increase the rate of densely packed occlusion and reduce the long-term recanalization rate in the embolization of large aneurysms.^5^^,^^6^

Yoshikazu et al^7^ compared the effects of standard platinum coil (GDC) and HydroCoil on a canine model of a large-sized, wide-necked, high-flow bifurcation aneurysm. The packing ratios of the HydroCoil-treated group and the GDC-treated group were 75.4% and 39.6%. Five of the six aneurysms (HydroCoil-treated group) were 100% and the other one was 90% embolized and remained stable 14 days and 3 months after surgery. However, two of the three aneurysms (GDC-treated group) underwent recanalization on postoperative 14th day and deteriorated 3 months after surgery. Thicker neointimal tissues were found at the aneurysm necks of HydroCoil-treated group. HydroCoil could also lower the risks of recanalization.^7^ Currently, HES has been clinically applied in the embolization of intracranial aneurysm.^3^^-^^6^^,^^8^^,^^9^

Arthur et al^4^ retrospectively analyzed the 33 aneurysms of 30 patients receiving HES treatment. All the patients had been treated for at least 6 months prior to data analysis. The clinical records and angiographic follow-up were reviewed. They concluded that HES significantly facilitated neointimal formation across the neck of the treated aneurysms with few complications. They also primarily verified that HES could safely and effectively treat aneurysms and may allow an improved aneurysm filling. Deshaieset al^10^ reported the 1-year recurrence and complication rates of patients embolized with HES. The safe HES provided outstanding 1-year occlusion for small- and large-sized aneurysms.

In our study, the embolization with HydroCoil led to more densely packed occlusion cases (27 cases, 54%) than that with ordinary coils (15 cases, 30%). The packing density increases exponentially until HES completely expands in human body (20 min). The volumes of the completely expanded HES Series 10, 14 and 18 are 5, 7 and 11 times larger than those of the same-length platinum coil Series 10. In other word, a less and shorter HES is able to provide exactly the same packing density.^2^ Wang et al^11^ reported that HydroCoil and ordinary coils did not differ significantly in the densely packed occlusion of small-sized aneurysm, whereas they differed significantly in embolizing large- and medium-sized aneurysms.

Thus far, most studies have demonstrated that HES can safely and potently embolize aneurysms with less recurrence and complications. Similarly, only one of the 27 cases receiving HydroCoil embolization suffered from aneurysm recurrence during the postoperative 2-year follow-up. Nevertheless, three and two of the 23 cases receiving ordinary coil embolization suffered from aneurysm recurrence. Although the increase of packing density can theoretically prevent aneurysm recurrence, HES has not been approved for clinical use until recently. Thus, follow-ups in a longer time span are still in need.

In summary, utilizing digital subtraction techniques (DSA, CTA) especially 3D imaging, the intracranial aneurysms images were analyzed, the 3D characteristics of the aneurysms, the positions of aneurysm necks and bodies, and the relationships between aneurysms and tumor-carrying artery or neighboring artery were clarified, the aneurysm spatial configuration was described, and different personalized assistance methods were designed to protect tumor-carrying arteries. Meanwhile, HES may function in curing large-sized, wide-necked, high-flow irregular aneurysms, reducing postoperative tumor resides and preventing recurrence due to the maximized packing intensity. Nevertheless, the results herein are lack of sufficient cases and long-term follow-up, thus further investigations are still needed.
